# Lumboperitoneal shunt and ventriculoperitoneal shunt for chronic hydrocephalus after aneurysmal subarachnoid hemorrhage: a comparison

**DOI:** 10.3389/fsurg.2024.1368493

**Published:** 2024-03-12

**Authors:** Xiaolei Li, Yuangang Wang, Bin Xia, Hongmin Che, Zhongnan Yan

**Affiliations:** Department of Neurosurgery, Xi’an Gaoxin Hospital, Xi’an, Shaanxi, China

**Keywords:** chronic hydrocephalus, aneurysmal subarachnoid hemorrhage, ventriculoperitoneal shunt, lumboperitoneal shunt, efficacy

## Abstract

**Objective:**

Chronic hydrocephalus after aneurysmal subarachnoid hemorrhage (aSAH) results in poor neurological outcomes and cognitive deficits. Currently, the main treatments for chronic hydrocephalus include ventriculoperitoneal shunt (VPS) and lumboperitoneal shunt (LPS); however, the optimal treatment for chronic hydrocephalus after aSAH remains controversial.

**Method:**

The records of 82 patients were retrospectively analyzed, and the patients were divided into VPS and LPS groups based on surgical methods. The efficacy, shunt successful rate and complications were compared. The assessments of treatment efficacy included the Evans index score (EIS), Keifer's hydrocephalus score (KHS), Mini-Mental State Examination (MMSE) score and functional independence measure (FIM). Patients were followed up for three months to observe the postoperative curative effects and complications.

**Results:**

The rate of shunt obstruction was significantly higher in the LPS group than that in the VPS group (*p* < 0.05), and the shunt successful rate was significantly higher in the VPS group than that in the LPS group (*p *< 0.05). The total rate of complications was 24.4% for LPS and 39% for VPS. The improvements in EIS, KHS, MMSE, and FIM within each group after the shunt were significantly different compared to those before shunt (*p *< 0.05). Compared to those in the LPS group, the improvements in EIS, KHS, MMSE, and FIM were significantly different in the VPS group after shunt (*p *< 0.05).

**Conclusion:**

Compared with LPS, VPS in the treatment for chronic hydrocephalus after aSAH had greater therapeutic efficacy, as indicated by improved radiological outcomes, improved shunt successful rate, improved clinical outcomes, and improved quality of life. Therefore, we believe that VPS is the preferred treatment option for chronic hydrocephalus after aSAH, while LPS should only be used as an alternative to VPS.

## 1 Introduction

Aneurysmal subarachnoid hemorrhage (aSAH) is a cerebrovascular disease characterized by bleeding in the subarachnoid space resulting from the rupture of an intracranial aneurysm. It is a life-threatening condition with a high incidence rate of 2–32 cases per 100,000 populations annually and is more commonly observed in individuals older than 50 years (1, 2). Although the occurrence of aSAH is comparatively lower than that of other cerebrovascular diseases, delayed neurological dysfunction develops in 30%–50% of aSAH survivors, leading to reduced quality of life (QOL) and augmented socioeconomic burden (3).

Hydrocephalus is a common complication in patients with aSAH, with an incidence ranging from 18% to 64%, depending on the patient's clinical condition (4). Hydrocephalus is classified as acute, subacute, or chronic based on the onset time course, corresponding to three stages: acute (24–72 h after aSAH) with an incidence of 20%, subacute (between days 4 and 13) with an incidence of 2%–3%, and chronic (after day 14) with an incidence of 7%–48% (5). The drainage of subarachnoid blood after aSAH plays a crucial role in preventing early and delayed brain injury. However, it is challenging to completely remove subarachnoid hemorrhage. Continuous drainage of cerebrospinal fluid is an important factor in facilitating subarachnoid blood drainage (6). External ventricular drainage is the most commonly used method to promote blood drainage from the subarachnoid space or ventricular system. Although it has a short duration and a low clearance rate of bloody cerebrospinal fluid, it is the preferred method for relieving acute hydrocephalus (7). Ventricular and lumbar drainage can enhance cerebrospinal fluid circulation and facilitate the elimination of toxic substances in bloody cerebrospinal fluid (8). Previous studies have shown that acute hydrocephalus is caused by blockage of cerebrospinal fluid (CSF) outflow from the ventricular system, whereas chronic hydrocephalus is associated with arachnoid granulations and fibrosis of the leptomeninges caused by aSAH. Clinically, acute hydrocephalus is typically treated with external ventricular drainage, and chronic hydrocephalus is mostly treated with shunted CSF drainage (3, 9).

Chronic hydrocephalus is a prevalent complication after aSAH and imposes heavy burdens on patients, such as prolonged hospital and intensive care unit stays, increased neurological morbidity, suboptimal neurological outcomes, cognitive impairments, and reduced quality of life. Currently, the primary treatment for chronic hydrocephalus involves CSF shunt placement, including the ventriculoatrial shunt, the ventriculoperitoneal shunt (VPS) and the lumboperitoneal shunt (LPS) (10, 11). VPS is the most widely accepted and used treatment option for chronic hydrocephalus after aSAH, although it has many complications, such as intracranial hemorrhage, shunt infection, excessive drainage, and seizure disorders. However, more recent studies have shown that LPS is the superior choice for chronic hydrocephalus after aSAH because it has several advantages, especially avoiding brain damage (12). In this regard, LPS has become the first-line and most commonly used treatment for idiopathic normal pressure hydrocephalus (INPH) in Japan (13). There is still debate about the best treatment for chronic hydrocephalus after aSAH (14, 15). Therefore, we conducted a retrospective analysis of the efficacy of LPS and VPS for the treatment of chronic hydrocephalus after aSAH. The purpose of this study was to provide evidence for the optimal option for chronic hydrocephalus after aSAH.

## 2 Materials and methods

### 2.1 Patients and eligibility

We consecutively and retrospectively analyzed the hospital records of 82 patients with chronic hydrocephalus after aSAH between January 2017 and May 2022, which were divided into the VPS group (41) and the LPS group (41) according to the treatment. The protocol of the study was conducted with the approval of the ethics committee of Xi'an Gaoxin Hospital. The programmable pressure valve shunt catheters used in the LPS group were obtained from Sophysa (Orsay, France), whereas those used in the VPS group were obtained from Medtronic (Minneapolis, USA).

The inclusion criteria were (1) an age ≥ 18 years; (2) chronic hydrocephalus confirmed by brain computed tomography (CT) and clinical manifestations. Hydrocephalus may manifest as headache, nausea, vomiting, coma, and/or gradual slowing of knowledge and motor activity, gait ataxia, cognitive impairment, and urinary incontinence (16); (3) more than two weeks after aneurysm clipping or interventional embolization; (4) VPS or LPS performed. The exclusion criteria were (1) an age < 18 years; (2) lost to follow-up; (3) history of previous hydrocephalus or history of shunt surgery; (4) without hydrocephalus after surgery for ruptured aneurysms and subarachnoid hemorrhage, or with temporary external drainage of acute hydrocephalus that was cured within two weeks; (5) with severe impairment of consciousness prior to shunt implantation; (6) obstructive hydrocephalus and asymptomatic hydrocephalus (Obstructive hydrocephalus not suitable for LPS, and a portion of asymptomatic hydrocephalus without specific treatment); (7) pregnant or nursing women; (8) with hepatic or renal insufficiency and pre-existing autoimmune diseases. The inclusion and exclusion criteria were the same for both groups.

### 2.2 Surgical procedure

In the VPS group (12): The patients were placed in the supine position with the head tilted to the contralateral side by 30°–45°, and an incision of approximately 3.0 cm was made at 6–7 cm above the patient's external occipital protuberance, with a midline point opened 3.0 cm beside the midline. The neurosurgeon drilled into the patient's skull, incised the patient's dura, and implanted the shunt tube along the parallel sagittal plane, aligned with the superciliary ridge. A 3.0 cm incision was made behind the ear, the shunt tube was guided into the incision through the subcutaneous tunnel, and the incision was subcutaneously enlarged to place the adjustable pressure shunt. An abdominal incision was made right next to the subxiphoid process and a subcutaneous tunnel was made along the patient's neck and chest to the incision. The shunt tube was inserted into the abdominal cavity through the subcutaneous tunnel, and the abdominal incision was sutured.

In the LPS group (12): The patients were placed in the lateral recumbent position with knees and neck bent, and then the lumbar intervertebral space of L3–4 or L4–5 was positioned as the puncture point. The neurosurgeon made a local skin incision of approximately 3.0 cm, and then inserted the beveled puncture needle into the lumbar intervertebral space and pulled out the puncture needle core after an obvious breakthrough sensation. When the CSF flowed out smoothly, the shunt tube was implanted into the lumbar cistern through the puncture needle sheath (with a depth of 5–8 cm). An approximately 3.0 cm incision was made at the upper end of the iliac crest, the shunt tube was guided into the incision through the subcutaneous tunnel, and the incision was subcutaneously enlarged to place the adjustable pressure shunt value. The neurosurgeon selected the anti-McBurney point as the abdominal incision (approximately 3.0 cm), and the abdominal section of the shunt tube was guided into the incision at the upper iliac crest through the subcutaneous tunnel. The distal and proximal shunts and the adjustable pressure shunt were connected in the prescribed direction, and the skin incision was sutured.

Before shunt, intracranial pressure was measured by lumbar puncture and initial value of the adjustable pressure shunt value was set according to intracranial pressure. Two types of implantations were performed by the same group of neurosurgeons with extensive surgical experience.

### 2.3 Data collection

The baseline information and follow-up data will be collected by at least two experienced and practiced assessors. The baseline and clinical characteristics studied included age, sex, BMI, hypertension, diabetes mellitus, hyperlipemia, operative methods of aSAH (clipping or embolization), time from onset to shunt surgery (days), operative time (hours), complications, and Evans index score (EIS), Keifer's hydrocephalus score (KHS), Mini-Mental State Examination (MMSE), and functional independence measure (FIM) before shunt surgery, 5 days after shunt surgery and 3 months after shunt surgery.

#### 2.3.1 Complications assessment

Complications including intracranial hemorrhage, shunt infection, seizure disorder, excessive drainage, shunt obstruction, radicular pain, and abdominal discomfort were recorded in detail after shunt. Since some patients had multiple complications, the total rate of complications was replaced by the proportion of patients with complications. According to previous studies, shunt failure was defined as the occurrence of clinical or radiologic signs or symptoms of shunt infection, obstruction, or malfunction requiring shunt revision after surgery. Shunt success was defined as the absence of shunt failure or good control of hydrocephalus without revision (17).

#### 2.3.2 Radiologic assessment

Radiologic assessment was performed using the Evans index score (EIS). EIS = maximum distance between the anterior horns of the lateral ventricles at the same horizontal level on cranial computed tomography (CT)/maximum distance between the largest intracranial plates at the same level. EIS ≥ 0.3 indicated hydrocephalus (18). EIS was reviewed via CT, and recorded before shunt, 5 days after shunt and 3 months after shunt.

#### 2.3.3 Clinical symptoms assessment

Keifer's hydrocephalus score (KHS) was applied to assess the improvement in clinical symptoms in the VPS and LPS groups before shunt, 5 days after shunt and 3 months after shunt. KHS was determined on a scale of 1–5 in 5 areas: gait disturbance, mental disorder, urinary incontinence, headache, and vertigo. The 5 scores were summed, and the lower the score, the better the improvement (17, 19).

#### 2.3.4 Cognitive function assessment

The Mini-Mental State Examination (MMSE) score was used to assess the cognitive dysfunction in the VPS and LPS groups before shunt, 5 days after shunt and 3 months after shunt. The total score of MMSE was 30, with 26–30 as normal, 21–25 as mild dementia, 10–20 as moderate dementia, and 0–9 as severe dementia. The lower score indicated a more serious neurological deficit (20).

#### 2.3.5 QOL assessment

The functional independence measure (FIM) was used to evaluate the QOL in the VPS and LPS groups before shunt, 5 days after shunt and 3 months after shunt, with a total score of 126. The lower score after evaluation indicated worse self-care ability (21).

### 2.4 Statistical analysis

SPSS22.0 software (IBM, Armonk, NY, USA) was used for date entry and statistical analysis, and GraphPad Prism 8.0 was used for image processing. After confirming the distribution, Student's unpaired *t*-test was used for intergroup data that conformed to a normal distribution, and the Mann-Whitney *U* test was used for intergroup data that conformed to a non-normal distribution. The quantitative data are presented as the mean ± standard deviation, whereas the qualitative data are presented in terms of frequency or percentage (%), and comparisons between groups were performed using the *χ*^2^ or chi-square test. Repeated measures two-way ANOVA was used to compare EIS, KHS, MMSE, and FIM scores before shunt surgery, 5 days after shunt surgery and 3 months after shunt surgery between the VPS and LPS groups. A shunt successful rate curve was generated using the Kaplan‒Meier method, and the log-rank test was used to compare the differences between the two groups. Statistical significance was set at *p *< 0.05. significance. *p < 0.05, **p < 0.01, ***p < 0.001, ****p < 0.0001, ns (not significant) *p *> 0.05.

## 3 Results

### 3.1 Comparison of baseline data between the two groups

The data from 82 (57 males and 45 females) patients, who were treated with VPS or LPS for chronic hydrocephalus after aSAH, were consecutively and retrospectively analyzed. Parameters such as age, sex, BMI, hypertension, diabetes, hyperlipemia, operative methods of aSAH, time from onset to shunt surgery, and EIS, KHS, MMSE, and FIM before shunt did not significantly differ between the LPS group and the VPS group (*p *> 0.05; Table 1). However, compared with those in the VPS group, then operative times in the LPS group were shorter (*p *< 0.001; Table 1).

**Table 1:** Comparison of baseline data between the two groups.

Variables	LPS (*n *= 41)	VPS (*n *= 41)	Total (*n *= 82)	Test value	*p*-value
Age (years)	56.68 ± 6.62	56.34 ± 6.81	56.51 ± 6.67	−0.23	0.818
Sex [*n* (%)]				0.868	0.352
Male	29 (70.7)	25 (61)	54 (65.9)		
Female	12 (29.3)	16 (39)	28 (34.1)		
BMI (kg/m^2^)	23.99 ± 1.44	24.30 ± 1.53	24.14 ± 1.48	−1.21	0.227
Hypertension	33 (80.5)	32 (78)	65 (79.3)	0.074	0.785
Diabetes	21 (51.2)	18 (43.9)	40 (48.8)	0.440	0.507
Hyperlipemia	15 (36.6)	13 (31.7)	28 (34.1)	0.217	0.641
Operative methods of aSAH [*n* (%)]				0.841	0.359
Clipping	17 (41.5)	18 (43.9)	35 (42.7)		
Embolization	24 (58.5)	23 (56.1)	47 (57.3)		
Time from onset to shunt surgery (days)	36.44 ± 5.98	35.15 ± 6.44	35.79 ± 6.21	0.942	0.349
Operative time (hours)	62.49 ± 9.0	86.22 ± 9.16	74.35 ± 14.96	11.836	<0.001
Before shunt					
EIS	0.354 (0.347, 0.358)	0.354 (0.349, 0.358)	0.354 (0.348, 0.358)	−0.381	0.703
KHS	19 (18, 20.5)	19 (18, 21)	19 (18, 21)	−0.656	0.512
MMSE	14 (11, 15)	13 (11, 17)	13.5 (11, 16)	−0.177	0.859
FIM	18 (16.5, 22)	19 (17, 23.5)	19(17, 22.25)	−0.698	0.485

### 3.2 Comparison of complications after shunt surgery

Complications were observed in 10 patients (24.4%) in the LPS group and 16 patients (39%) in the VPS group. The total rate of complications was higher in the VPS shunt group than in the LPS group (*p *> 0.05). There were 5 cases of intracranial hemorrhage in the VPS group, which was more than that in the LPS group (*p *> 0.05). Shunt infections were also more frequent in the VPS group, with 3 cases reported, than in the LPS group (*p *> 0.05). Seizure disorders occurred more frequently in the VPS group than in the LPS group, with 3 cases reported (*p *> 0.05). Additionally, 3 patients in the VPS group had excessive drainage, which was higher than that in the LPS group (*p *> 0.05). In addition, 2 patients in the VPS group reported abdominal pain, which was higher than that in the LPS group (*p *> 0.05). Interestingly, one LPS-treated patient experienced significant radicular pain, which was greater than that in the VPS group (*p *> 0.05). Although the comparison of these complications was not statistically significant, it could provide some indications that LPS had a lower complication rate than VPS. Shunt obstruction was observed in 6 patients in the LPS group, which was significantly greater than that in the VPS group (*p *< 0.05; Table 2). There was no significant difference in the total rate of complications between the two groups (*p *> 0.05; Table 2).

**Table 2:** Comparison of complications after treatment in the two groups.

Parameter [*n* (%)]	LPS (*n *= 41)	VPS (*n *= 41)	*χ* ^2^	*p*-value
Intracranial hemorrhage	1 (2.4)	5 (12.20)	2.877	0.09
Shunt infection	1 (2.4)	3 (7.3)	1.051	0.305
Seizure disorder	1 (2.4)	3 (7.3)	1.051	0.305
Excessive drainage	0	3 (7.3)	3.114	0.078
Shunt obstruction	6 (14.6)	1 (2.4)	3.905	0.048
Radicular pain	1 (2.4)	0	1.012	0.314
Abdominal discomfort	1 (2.4)	2 (4.9)	0.346	0.556
Total rate of complications	10 (24.4)	16 (39)	2.027	0.154

### 3.3 Comparison of the shunt successful rate between the two groups after surgery

**Figure 1:**
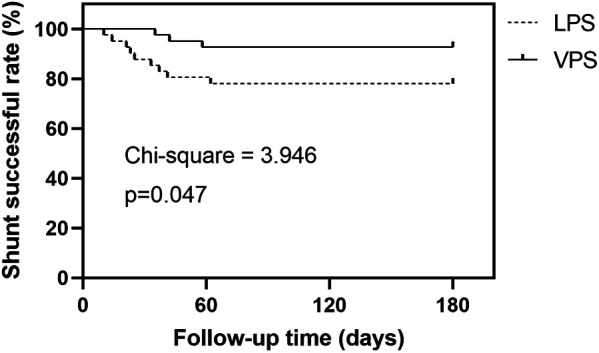
Kaplan-Meier survival analysis was used to compare the shunt successful rates of the two groups.

All patients who required shunt revision had shunt revision within three months of follow-up. In the LPS group, six patients had shunt obstruction due to end-of-abdomen displacement, one patient had shunt infection due to abdominal infection, and two patients had shunt malfunction. In comparison, among the patients in the VPS group who underwent shunt revision, one patient experienced shunt obstruction caused by intracranial hemorrhage. Additionally, two patients developed shunt infection as a result of intracranial infection. It is worth noting that the remaining patient with shunt infection did not require shunt revision due to symptomatic improvement following anti-infective therapy. The Kaplan-Meier curve was used to compare the shunt successful rates of the two groups. The log-rank test was employed to test the shunt successful rates of shunting in the two groups. The success rate of VPS is significantly higher than that of LPS (*p *< 0.05; Figure 1).

### 3.4 Comparison of radiological data and EIS between the two groups

**Figure 2:**
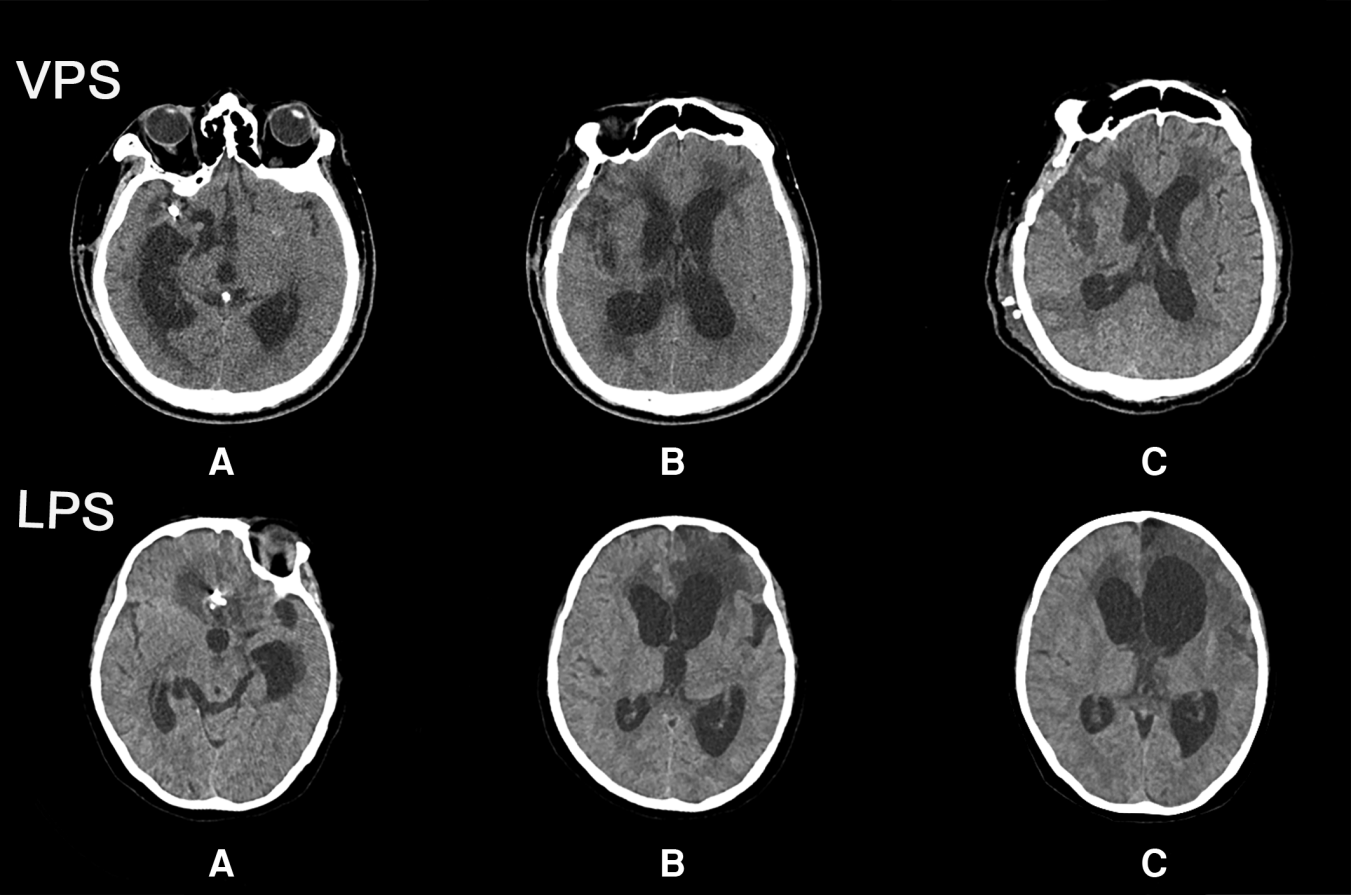
CT observing the changes of hydrocephalus before and after shunt between the two groups. (**A**) Brain CT showing the location of intracranial aneurysms and the surgical methods of intracranial aneurysms. (**B**) Brain CT showing hydrocephalus after intracranial aneurysm surgery. (**C**) Brain CT showing ventricles after hydrocephalus surgery.

**Figure 3:**
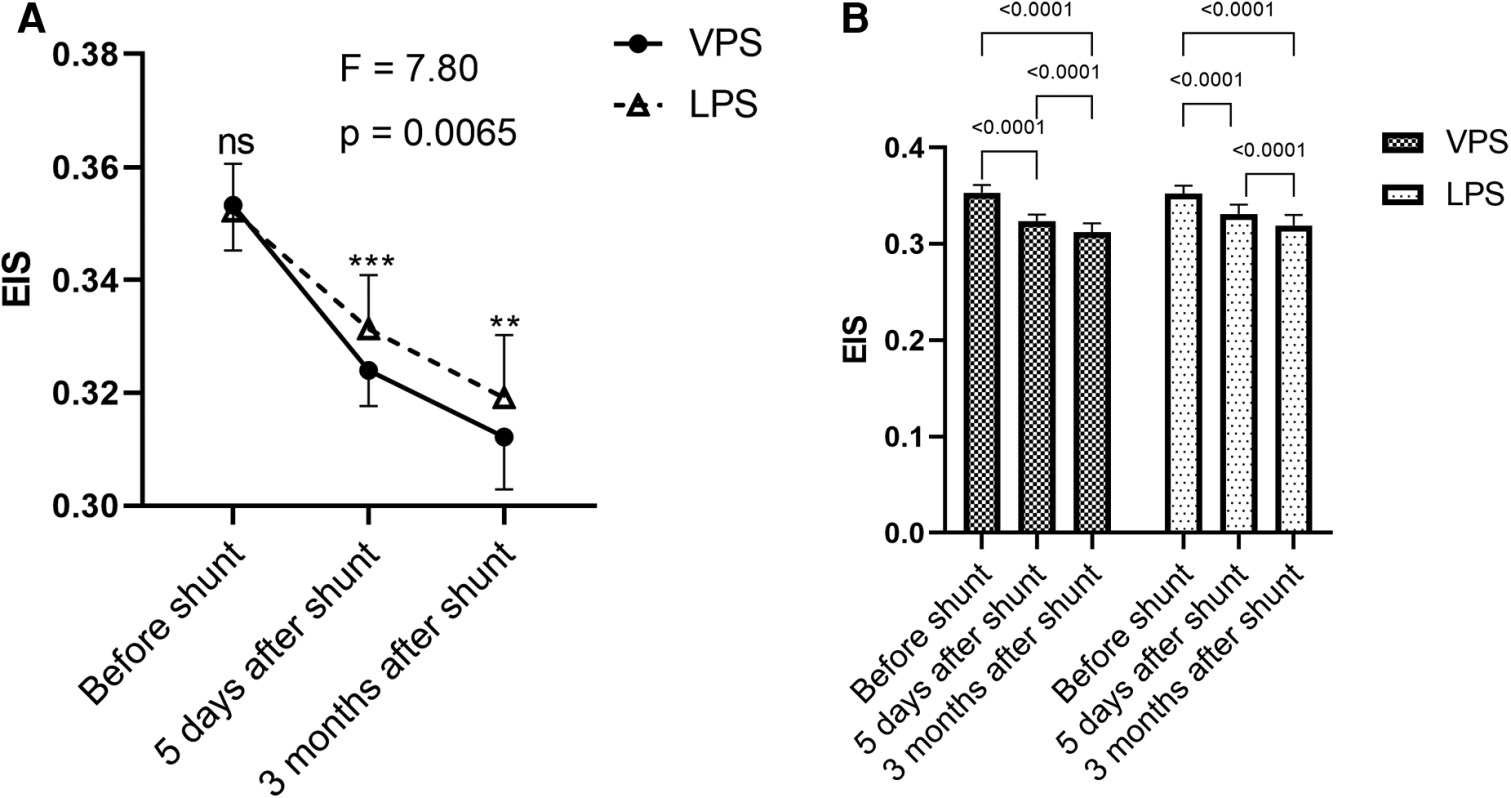
Comparison of EIS in the two groups before and after shunt. (**A**) Comparison of before shunt and after shunt EIS between the two groups. (**B**) Comparison of EIS between before shunt and after shunt in the two groups.

The majority of patients with hydrocephalus improved radiologically after shunt surgery, but some patients had enlarged ventricles in the two groups (Figure 2). In the VPS group, EISs of 5 days after shunt and 3 months after shunt were lower than those of before shunt, and EIS of 3 months after shunt was lower than that of 5 days after surgery, with statistical significance. In LPS group, EIS of 5 days after shunt and EIS of 3 months after shunt were lower than those of before shunt, and EIS of 3 months after shunt was lower than that of 5 days after surgery, with statistical significance. EIS of 5 days and 3 months after shunt in VPS group were lower than those in LPS group, and the difference was statistically significant. There was a statistically significant difference in EIS between the two groups according to repeated-measure ANOVA (*p *= 0.0065; Figure 3).

### 3.5 Comparison of clinical symptoms in the two groups before and after shunt

**Figure 4:**
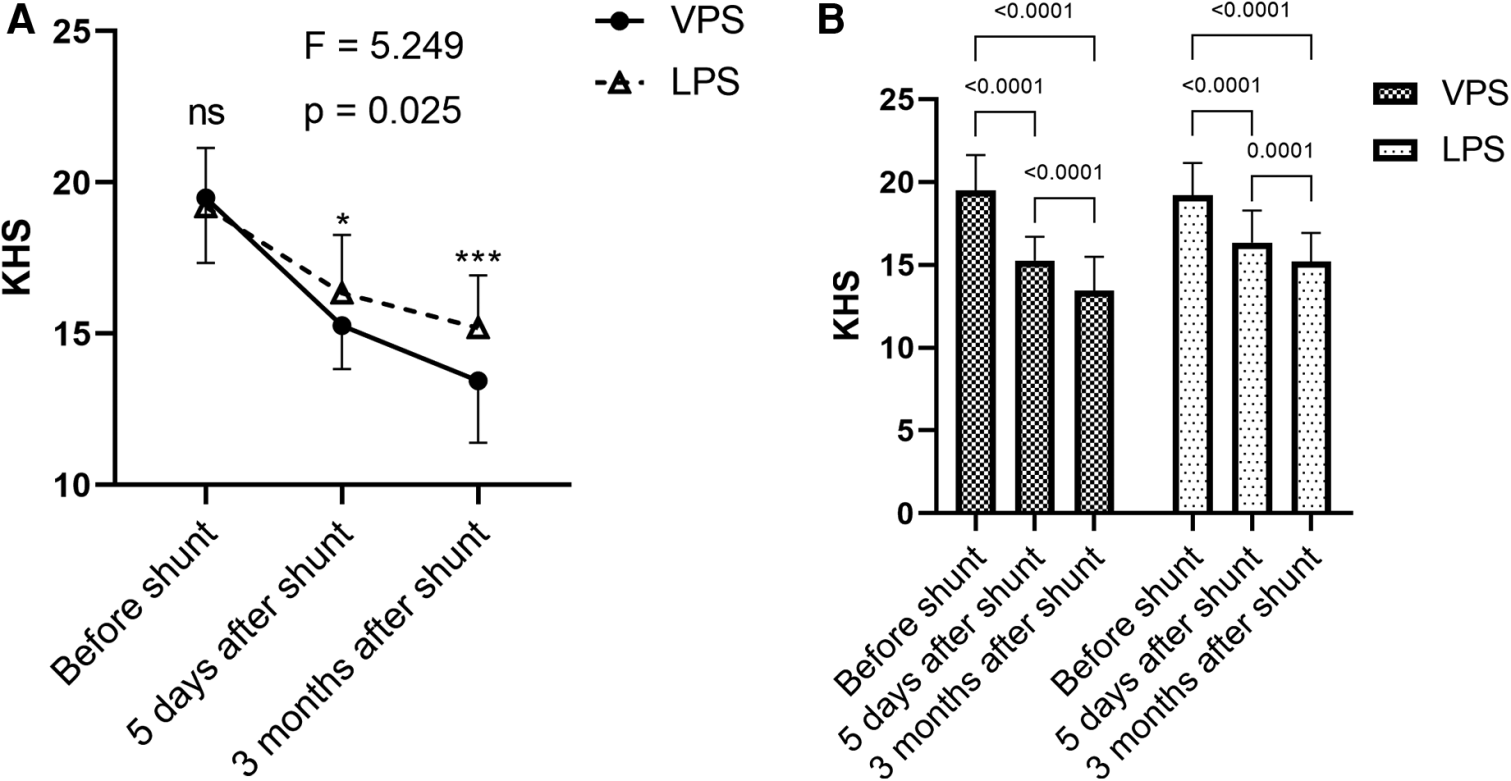
Comparison of KHS in the two groups before and after shunt. (**A**) Comparison of before shunt and after shunt KHS between the two groups. (**B**) Comparison of KHS between before shunt and after shunt in the two groups.

Compared with those before shunt, the clinical symptoms of each group were significantly improved at 5 days and 3 months after shunt surgery, and the improvement in clinical symptoms at 3 months after shunt surgery was more significant than that at 5 days after shunt surgery, the differences were statistically significant. Compared with those in the LPS group, KHSs in the VPS group was significantly lower, and KHS in the VPS group were both significantly lower at 5 days and 3 months after shunt surgery, indicating that VPS was more effective in improving the clinical symptoms of patients with chronic hydrocephalus after aSAH (Figure 4). There were statistically significant differences in KHS between the two groups (*p *= 0.025; Figure 4A), and there were statistically significant differences in KHS between the two groups at 5 days after (*p *< 0.05) shunt and 3 months after shunt (*p *< 0.001). There were statistically significant differences in KHS between points of time within each group (*p *< 0.0001; Figure 4B).

### 3.6 Comparison of cognitive function in the two groups before and after shunt

**Figure 5:**
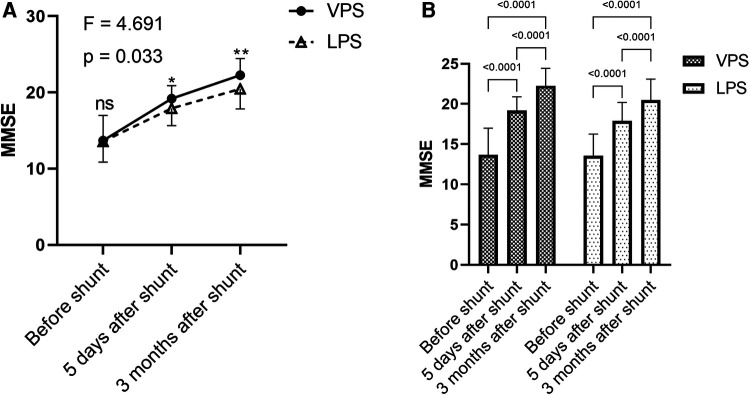
Comparison of MMSE in the two groups before and after shunt. (**A**) Comparison of before shunt and after shunt MMSE between the two groups. (**B**) Comparison of FIM between before shunt and after shunt in the two groups.

There was no significant difference in the MMSE between the two groups before surgery (*p *< 0.05). After surgery, the MMSE scores of the two groups improved and were greater than those before surgery (*p *< 0.0001), and MMSE 3 months after surgery was greater than that 5 days after surgery (*p *< 0.0001; Figure 5B). Compared to the cognitive between the two groups, MMSE of the VPS group was higher than that of the LPS group (*p *= 0.033; Figure 5A), and MMSEs of the VPS group after surgery were higher than those of the LPS group after surgery at the same time point, while these were statistically significant (*p *< 0.05; Figure 5A).

### 3.7 Comparison of QOL in the two groups before and after treatment

**Figure 6:**
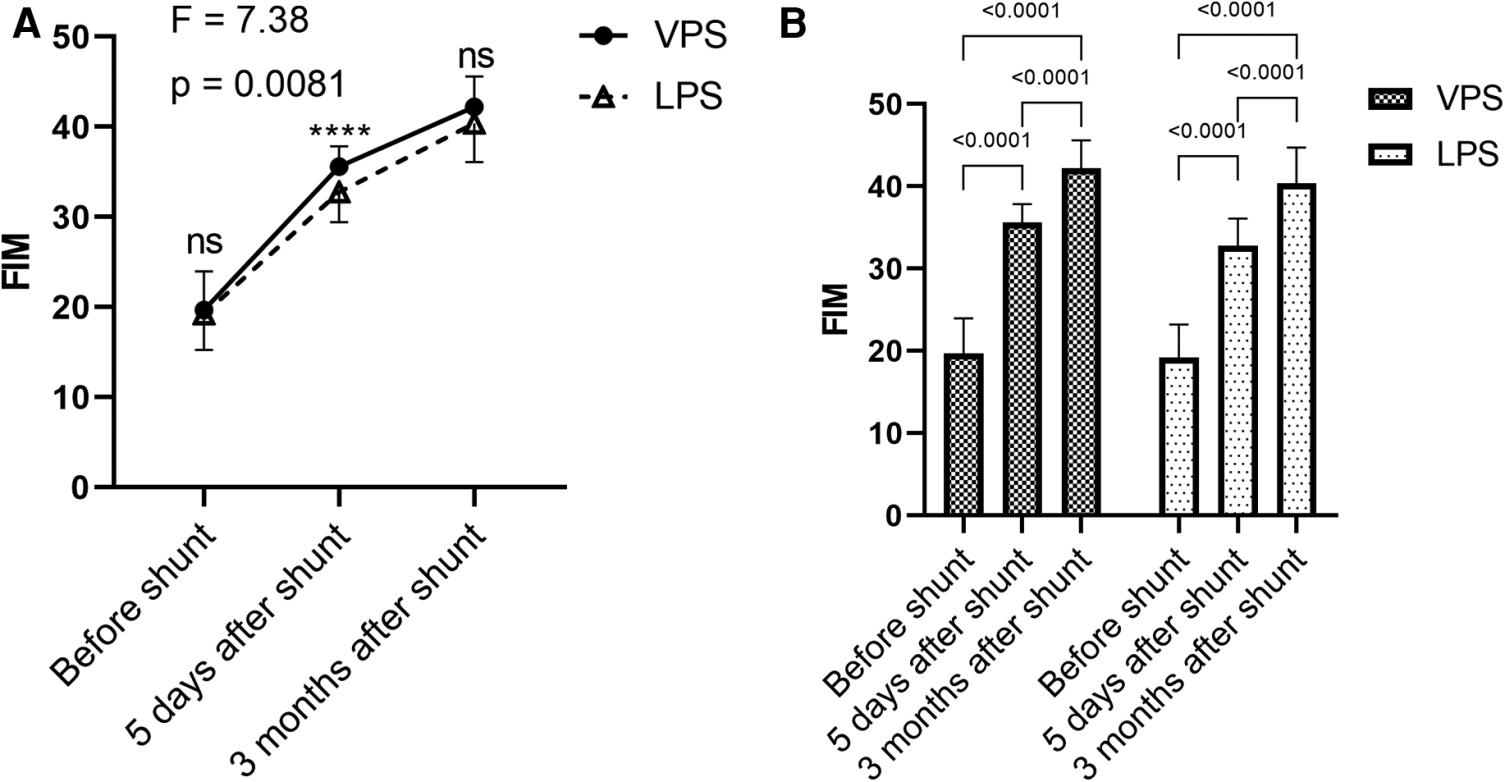
Comparison of FIM in the two groups before and after shunt. (**A**) Comparison of before shunt and after shunt FIM between the two groups. (**B**) Comparison of FIM between before shunt and after shunt in the two groups.

FIM was applied to compare the improvements in the QOL. Between the two groups, there was no significant difference in FIM before shunt and 3 months after shunt (*p *> 0.05), but there was significant difference in FIM 5 days after shunt (*p *< 0.0001). There was significant difference in FIM between the two groups (*p *= 0.0081; Figure 6A). After shunt, FIM in the two groups were improved, and were higher than those before surgery, while there were statistically significant differences in FIM between time points within each group (*p *< 0.0001; Figure 6B).

## 4 Discussion

Chronic hydrocephalus after aneurysm subarachnoid hemorrhage (aSAH) can result in poor neurological outcomes and cognitive deficits, and is a well-known complication of ruptured intracranial aneurysms (22). Currently, the standard treatment for chronic hydrocephalus remains cerebrospinal fluid shunting, which transfers accumulated cerebrospinal fluid to the peritoneal cavity and involves the permanent placement of VPS and LPS; however, previous studies had shown that 59% of patients with hydrocephalus benefit from shunt surgery at the 1-year follow-up, and 29% of those patients show significant improvement. For the past two decades, VPS has been the dominant surgical treatment for hydrocephalus due to its high success rate (23). On the other hand, LPS has received increasing attention from neurosurgeons due to several potential advantages, including the lack of need to enter the ventricle to avoid brain tissue damage, a decreased risk of postoperative infection, and convenient operation (24).

According to previous studies, the rate of VPS complications, including hemorrhage, infection, excessive drainage, and seizure disorder, ranged from 17%-33%, and was higher than that of LPS complications (25). Aoki's team concluded that LPS should be given greater consideration for patients with communicating hydrocephalus, especially after aSAH, because the rate of complications was significantly lower in the LPS group than in the VPS group (26). However, these studies only compared complication rates between the two groups and did not consider improvements in clinical practice, radiology or quality-of-life. In our study, we demonstrated that the total rate of complications in the VPS group was higher than in the LPS group, but there was no significant difference. Whereas, the rate of shunt obstruction was 14.6% in the LPS group, which was significantly higher than 10.8% reported by Vajpeyi et al. In the VPS group, the rate of shunt obstruction was 2.4%, which was in agreement with the range of 0%-13.8% as described by Singh and Yadav (27). The rate of shunt obstruction in the LPS was higher than in the VPS, and there was significant difference, which was consistent with the result reported by Lund-Johansen et al. (28). The high rate of shunt obstruction in the LPS group was strongly associated with shunt failure, which was an unacceptably common outcome. Miyajima et al. suggested that patients treated with LPS had a higher rate of shunt failure requiring shunt revision and a lower efficacy than patients treated with VPS at 1 year after shunt (29). In our study, 6 patients failed within 3 months due to shunt obstruction, and between the two groups, the shunt successful rate in the VPS group was significantly higher than that in the LPS group.

We characterized post-operative patients’ improvement in four aspects: radiology, clinical, cognitive function, and quality of life. The EIS was used as a quantitative indicator to describe radiologic improvement. We found that after shunt, the EIS in the two groups decreased significantly compared to before surgery, and gradually improved over time in radiologic aspect. However, there was a statistically significant difference in the EIS between the two groups, which was consistent with the results reported by S.Kang (30). Because there was no significant retraction of the ventricles on postoperative imaging, neurosurgeons and patients considered LPS to be a fewer effective procedure. Previous studies have demonstrated that both LPS and VPS improve clinical symptoms and have consistent clinical outcomes in the treatment of idiopathic normal pressure hydrocephalus (31). In our study, the KHS was applied to compare improvements in clinical symptoms. The KHS increased after surgery in both groups, indicating that shunt could improve clinical symptoms. There were differences in KHS between before shunt, 5 days after shunt and 3 months after shunt within each group, indicating that the clinical symptoms improved more significantly with time, but there were significant differences in KHS between the two groups, indicating that compared with LPS, VPS was more effective in improving the clinical symptoms of the patients with chronic hydrocephalus after aSAH. Chronic hydrocephalus after aSAH causes cognitive dysfunction in patients, which can be improved by shunt (32). The results of this study showed that the post-operative MMSE improved in the two groups, and the improvement was more significant over time, with significant differences between the two groups. The above results suggested that VPS and LPS can improve the patients' neurological function and cognitive dysfunction by reducing intracranial pressure, and the effect of VPS is more significant than that of LPS. Chronic hydrocephalus after aSAH affects the quality of life of patients (33). After LPS and VPS treatment, the FIM of both groups increased, indicating that the two shunts can improve the quality of life of patients, but there was a significant difference in the FIM between the two groups, suggesting that the VPS is more capable of improving the quality of life of patients compared with the LPS.

We hypothesized that the following reasons contributed to the high rate of shunt obstruction and poor efficacy in the LPS group. First, spinal canal arachnoid adhesions and inflammatory reactions caused by subarachnoid hematoma absorption in the spinal canal after aSAH resulted in poor CSF drainage. Second, patients with aSAH were bedridden or wheelchair-bound for long periods of time after surgery, which affects CSF dynamics. Third, patients with aSAH may require repeated lumbar punctures during treatment, resulting in localized spinal arachnoid adhesions or inflammatory reactions. Finally, there was an increase in protein levels or cell counts in the CSF after shunt. The reasons for this remain to be further studied and verified (34).

This study has several limitations. First, we analyzed surgical outcomes based on a single institution retrospective study in the presence of possible systematic bias and variation. Second, the patient population was relatively small and long-term data were lacking. Third, since LPS and VPS were two different surgical procedures and the types of catheters used were different, this also had some impact on shunt outcomes.

## 5 Conclusion

In our study, we found that, compared with LPS, VPS in the treatment for chronic hydrocephalus after aSAH had greater therapeutic efficacy, as indicated by improved radiological outcomes, improved shunt successful rate, improved clinical outcomes, and improved quality of life. Therefore, we believe that VPS is the preferred treatment option for chronic hydrocephalus after aSAH, while LPS can only be used as an alternative to VPS.

## Data Availability

The raw data supporting the conclusions of this article will be made available by the authors, without undue reservation.
